# Modifications in Glass Ionomer Cements: Nano-Sized Fillers and Bioactive Nanoceramics

**DOI:** 10.3390/ijms17071134

**Published:** 2016-07-14

**Authors:** Shariq Najeeb, Zohaib Khurshid, Muhammad Sohail Zafar, Abdul Samad Khan, Sana Zohaib, Juan Manuel Nuñez Martí, Salvatore Sauro, Jukka Pekka Matinlinna, Ihtesham Ur Rehman

**Affiliations:** 1 Department of Restorative Dental Sciences, Al-Farabi Colleges, P.O Box 361724, Riyadh 11313, Saudi Arabia; shariqnajeeb@gmail.com; 2 Department of Dental Biomaterials, College of Dentistry, King Faisal University, P.O. Box 400, Al-Hofuf 31982, Saudi Arabia; drzohaibkhurshid@gmail.com; 3 Department of Restorative Dentistry, College of Dentistry, Taibah University, Madina Munawwarrah 41311, Saudi Arabia; mzafar@taibahu.edu.sa; 4 Interdisciplinary Research Centre in Biomedical Materials, COMSATS Institute of Information Technology, Defence Road, off Raiwind Road, Lahore 54000, Pakistan; draskhan@ciitlahore.edu.pk; 5 Department of Biomedical Engineering, College of Engineering, King Faisal University, Al-Hofuf 31982, Saudia Arabia; szohaib@kfu.edu.sa; 6 Preventive and Minimally Invasive Dentistry (Spanish Course), Departamento de Odontología, Facultad de Ciencias de la Salud, Universidad CEU-Cardenal Herrera, Valencia 46115, Spain; juan.nunez@uch.ceu.es; 7 Dental Biomaterials, Preventive and Minimally Invasive Dentistry (Bilingual course), Departamento de Odontología, Facultad de Ciencias de la Salud, Universidad CEU-Cardenal Herrera, Valencia 46115, Spain; salvatore.sauro@uch.ceu.es; 8 The University of Hong Kong, Faculty of Dentistry, Dental Materials Science, Hong Kong, China; jpmat@hku.hk; 9 Department of Materials Science and Engineering, The Kroto Research Institute, The University of Sheffield, North Campus, Broad Lane, Sheffield S3 7HQ, UK

**Keywords:** glass ionomer cement, restorative dentistry, nanotechnology, adhesive dentistry

## Abstract

Glass ionomer cements (GICs) are being used for a wide range of applications in dentistry. In order to overcome the poor mechanical properties of glass ionomers, several modifications have been introduced to the conventional GICs. Nanotechnology involves the use of systems, modifications or materials the size of which is in the range of 1–100 nm. Nano-modification of conventional GICs and resin modified GICs (RMGICs) can be achieved by incorporation of nano-sized fillers to RMGICs, reducing the size of the glass particles, and introducing nano-sized bioceramics to the glass powder. Studies suggest that the commercially available nano-filled RMGIC does not hold any significant advantage over conventional RMGICs as far as the mechanical and bonding properties are concerned. Conversely, incorporation of nano-sized apatite crystals not only increases the mechanical properties of conventional GICs, but also can enhance fluoride release and bioactivity. By increasing the crystallinity of the set matrix, apatites can make the set cement chemically more stable, insoluble, and improve the bond strength with tooth structure. Increased fluoride release can also reduce and arrest secondary caries. However, due to a lack of long-term clinical studies, the use of nano-modified glass ionomers is still limited in daily clinical dentistry. In addition to the in vitro and in vivo studies, more randomized clinical trials are required to justify the use of these promising materials. The aim of this paper is to review the modification performed in GIC-based materials to improve their physicochemical properties.

## 1. Introduction

The concept of using synthetic biomaterials to replace lost or damaged tissue is not new [1,2]. For instance, plaster of Paris was pioneered as bone substitution around at the end of the 19th century. Dental silver amalgams are restorative materials that are still being used after more than 150 years [2,3]. A good example of modern dental materials is glass ionomer cement (GIC) that has revolutionized the restorative approaches, particularly in minimally invasive dentistry [4]. Bioactivity implies the induction of cellular growth, proliferation and tissue formation by a biomaterial. Additionally, bioactivity also signifies an anti-bacterial effect of a material to prevent or cure infection in the tissues. GICs contain alumino-fluorosilicate glasses which have inherent bioactive properties due to the presence of silicates and fluorides [3]. Each modification with any significant outcome has been considered in relation to effects on the final properties of GICs. In addition, the current status and future perspectives of nano-modified glass ionomers have been assessed.

**Figure 1:**
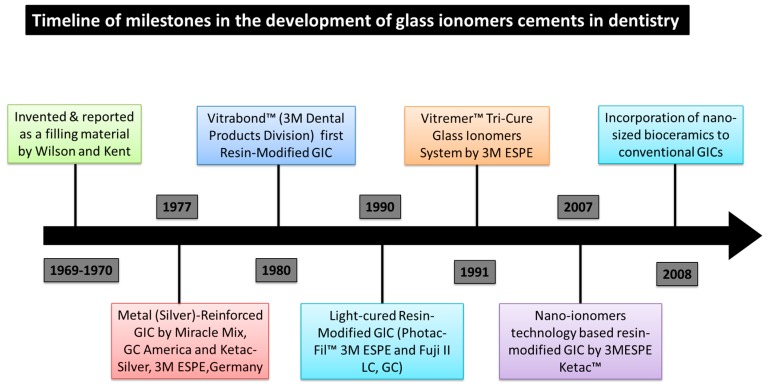
A timeline of the development and advances in glass ionomer cements (GICs). Originally invented as powder and liquid formulation by Wilson and Kent, GICs have undergone a number of modifications to improve their mechanical properties. More recently, nano-filled resin modified and conventional glass ionomers have been developed.

A dental material group, GICs, was originally invented by Wilson and Kent in 1969, and it was novel at the time of its invention. Several key benefits characterize GIC, such as its ability to bind chemically with tooth structures via chelation of carboxyl group of acid polymeric chains and calcium ions (Ca^2+^) in the apatite of enamel and dentine [5]. In addition, GICs have acceptable translucency, color and may exert an anti-carious effect due to release of fluoride (F^−^) ions [6,7,8,9]. Since the invention of GIC, further research continued to improve its properties further. Figure 1 shows the key milestones in the development of GICs.

**Figure 2:**
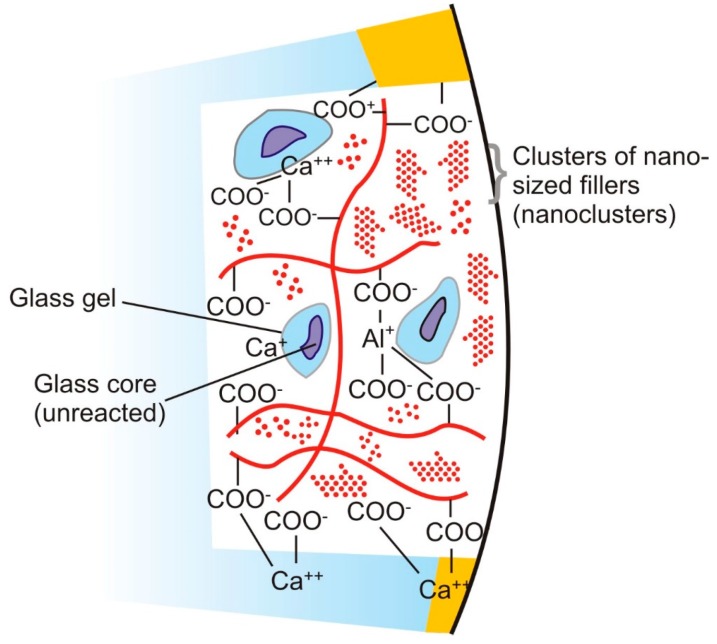
The structure of set glass ionomer cement and mechanism of bonding with tooth structure. The structure consists of unset glass particles which are ionically bonded to the cross-linked polyacrylate matrix via a gelation reaction. Bonding is achieved by the ionic interaction of the carboxylate groups (-COO^−^) with the apatite crystals present in the tooth structure. In nano-filled ionomers, clusters of nano-sized fillers or bioceramics can be present embedded in the cement matrix.

Conventionally, GICs comprised of two main components: a powder of fluoro-aluminosilicate glass and aqueous solution containing polyalkenoic acids which are carboxylic acids [3,10,11,12,13,14]. Polyacrylic acid is the main constituent of the aqueous component. However, less viscous polyacids, such as maleic and itaconic acids may also be present in the solution so that manipulation is easier [15,16,17]. Other additives, such as Ba- and Sr-salts can be added to the powder to impart radiopacity. Tartaric acid is usually added to the liquid component to further enhance the handling properties and increase the working time [2,3]. Conventional glass ionomer cements set via an acid-base reaction between the polyacrylic acid and fluoro-aluminosilicate glass particles [3,14]. The initial setting reaction is a gelation reaction between the components [12], followed by binding of the unreacted glass particles that act as fillers in the silica (SiO_2_) gel matrix. Hardening of the resultant composite takes place due to the cross-linking of the polymeric chains of the polyacid component (cross-linked acrylate matrix) with calcium and aluminum ions present in the powder component. GICs set in 2–3 min, but the chemical reaction for complete hardening may occur during the following 48 h [2,3]. Usually, sodium and fluoride ions do not react chemically and remain unreacted within the matrix. The final “maturation” of the cement may take several months while the aluminum ions may be released slowly and binding of water by the acid and glass takes place [18]. However, a study by Zainuddin et al. suggests that aluminum may remain in the cement structure for up to one year [19]. Figure 2 illustrates the structure of the set GIC and the thought bonding mechanism.

Glass ionomer cements are being used for a wide range of applications in dentistry [14]. Due to their high fluoride release and white color, dental applications of GICs include restoration of deciduous teeth [20], anterior class III and V restorations [21,22], cementation (luting) of crowns, bridges, and orthodontic appliances [23,24,25], restorations of non-carious teeth with minimal preparation [26,27], temporary cementation of crowns and other indirect restorations [28], restoration of teeth via the sandwich technique [29,30], and as materials for atraumatic restorative therapy (ART) [31]. Indeed, minimally invasive dentistry can also be applied for the treatment of deeper carious lesions by adopting the atraumatic restorative treatment approach with the scope to selectively remove infected carious tissue leaving as much caries-affected dentin as possible for therapeutic remineralization [32,33]. A more critical problem associated with such aesthetic restorations is the absence of therapeutic remineralization of caries-affected dentin and the poor durability/integrity of the resin–dentin interface during aging [34]. Atraumatic restorative treatments are considered to have a combined technique-material effect. It requires removal of the caries-infected dental tissues in order to arrest caries and induces dental remineralization while utilizing the healing potential of glass ionomer cements [26,35]. In addition, due to their high bioactivity, GICs may also be used as bone cements [36]. As discussed in this review, research is being conducted to impart even more bioactive properties to GICs via the incorporation of bioactive materials such as various apatites and titanium dioxide. In contrast to “bioinert” materials such as resin composites, dental silver amalgam, and porcelain, bioactive restorations have a more positive effect on remineralization and tissue regeneration [37].

No dental material today has ideal properties for any dental application [1,2,3,38]. This said, GICs also carry a number of drawbacks such as brittleness, subsequently prone to fracture [39], poor wear resistance, and inadequate surface properties [40,41], as well as they are sensitive to moisture in the oral cavity when newly placed [42]. These said aspects restrict the use of GICs for many clinical situations. In order to overcome the poor mechanical properties of glass ionomers, several modifications have been introduced to the conventional GICs [43,44,45,46]. The key modifications include the combination of glass ionomer cements with auto-cured or photo-cured resin systems to produce resin-modified glass ionomer cements (RMGICs) [46,47]. Additionally, modification of GICs by incorporation polyvinyl phosphonic acid [48,49], fiber-reinforcement [50], bioactive apatite without [43] or with zirconia [51,52], zinc [53,54], strontium oxide [55], stainless steel [56], silica particles [57], amino acids [58], and *N*-vinylpyrrolidone [45] have all aimed at improving the mechanical and physical properties.

Nanotechnology involves the use of systems, modifications or materials which have the size in the range of 1–100 nm [59,60,61]. Key applications of nanotechnology in dentistry include implant surface modifications [62], production of reinforced polymeric composites by incorporation of nano-sized particles [60], and caries prevention [63]. Recent studies have suggested that incorporation of nano-sized particles or “nanoclusters” can improve the mechanical properties of dental restorative material such as resin composites [64,65,66]. Similar approaches have been attempted to improve the physical and mechanical characteristics of GIC using nanotechnology [43,44].

There are two approaches for the manufacture of nano-size particles: top-down and bottom-up [61]. The so-called top-down nanofabrication involves the production of nano-size particles by removing the bulk material. Some examples of top-down fabrication include are milling, machining, and lithography. On the other hand, the so-called bottom-up nanofabrication involves production of nano-sized particles atom by atom. Some examples of bottom-up nanofabrication are: tissue regeneration, protein synthesis, and biomimetic dental implant coatings. Production of nano-sized particles for incorporation to GICs, is manly carried out through top-down nanofabrication of bulk materials such as apatites, silicate glasses, and some metal oxides.

## 2. Powder-Modified Nano Glass Ionomers

It is well-documented that incorporation of nano-sized particles may improve the mechanical properties of polymeric dental materials [60,67]. De Caluwé et al. showed that doping conventional GICs with nano-sized glass particles can decrease the setting time and enhance the compression strength and elastic modulus [68]. The main advantages of decreasing setting times of direct restorative materials are e.g., enhanced ease of handling and manipulation. These decrease the treatment time, benefitting both the clinician as well as the patient. Enhancing the mechanical properties adds to the serviceability and self-life of restorative materials as they are able to withstand the masticatory and occlusal forces more efficiently. The process of mastication is quite complex as it involves forces in multiple directions. Therefore, owing to the quantitative nature of in vitro research, it is difficult to translate the results obtained in the laboratory to clinical practice.

Certain procedures, such as thermo-cycling are aimed at artificially aging restorative materials in the laboratory so the effect of oral temperature and moisture may be assessed. Given this, thermo-cycling has more deleterious effects on the mechanical properties of nano-filled GICs compared to conventional GICs. This may also compromise the long-term survival rate of such materials. However, in most studies, the in vitro testing of modified GICs against various cell-lines has been carried out, and none of them has reported the effect of such cements on animals. The chemical structure of glass ionomers can be mainly assessed by spectroscopic methods. Fourier transform infrared (FTIR) spectroscopy has been used to observe the effect of nano-modification of GICs via incorporation of apatite nano-crystals [44,45]. However, the effect of modification by using some other particles, TiO_2_ and ZrO_2_, has not been evaluated or reported.

As described above, in order to improve the mechanical, biological, and physical properties of powder-liquid formulations of GICs, various types of nano-size powders have been incorporated to the glass powder component. The mechanical properties have been summarized in Table 1.

### 2.1. Modification Using Nano-Apatite

Due to their chemistry being similar to that of mineralized bone and dental tissues, hydroxypatite and fluorohydroxyapatite have been used in many fields of dentistry such as implant dentistry [71], and caries prevention [59,72]. For instance, nano-hydroxyapatite (nHAp) crystals can favor remineralization of enamel [73,74]. Recently, resin composites modified by the addition of nHAp have been observed to have superior mechanical properties than unmodified resin composites [75,76]. Similarly, addition of nHAp or nano-fluoroapatite (nFAp) to the powder component of conventional GIC has a positive impact on the compressive, tensile, and flexural strengths of the set cement after being stored in distilled water for 7 days [44]. 

FTIR spectroscopy has revealed that addition of apatite to GIC powder increases the crystallinity of the set GIC, hence improving the chemical stability and water insolubility [44,45]. Such modifications result in better survival rates than commercially available GICs [44]. Since nFAp has lower solubility than nHAp or nFAp-containing GICs have better mechanical properties and bond strength compared to nHAp-containing GICs [44,45]. It has been suggested that the enhanced mechanical properties of apatite-modified GICs are the result of ionic interaction between the polyacrylic acid and the apatite crystals [45]. This enhancement is more pronounced when the nHAp-containing powder is added to polyacrylic acid, itatonic acid, and *N*-vinylpyrrolidone copolymers, instead of the standard polyacrylic copolymer. This has been attributed to the additional physiochemical interaction between the *N*-vinylpyrrolidone and the apatite crystals [45]. Moreover, nano-apatite containing glass ionomers are expected to have superior bonding to the tooth surface due to the possibility of the formation of the strong ionic linkages between the apatite crystals/particles in the cement and Ca-ions in the tooth structure [77]. Additionally, decreasing the particle size of apatites from micrometer scale to nanometer scale increases the surface area remarkably, and infiltration of the crystals into demineralised dentine as well as enamel pores; this may enhance bonding at the tooth-ionomer interface [78].

### 2.2. Modification with Nano-Sized HAp/Zr, CaF_2_ and TiO_2_ Particles

It has been recently reported by Gu et al. that the combined incorporation of HAp and zirconia (HAp/ZrO_2_) at concentrations of 4 vol. % to the GIC powder can improve the mechanical properties of the set GIC [52]. When specimens of set GIC were analyzed using scanning electron microscopy (SEM) a dense, uniform dispersion of the glass and HAp/ZrO_2_ particles within the matrix were revealed. This was the key factor for the enhancement of mechanical properties. However, more cracks were observed in the set structure possibly formed due to the weak ZrO_2_-glass interface when compared to unmodified GIC. Due to an apparently weak ZrO_2_-glass interface and a less proportion of the glass matrix, concentrations of HAp/ZrO_2_ exceeding 4 vol. % have a detrimental effect on the properties of the GIC [51].

Calcium fluoride (CaF_2_) nanoparticles can be incorporated into RMGICs to improve mechanical properties. However, this has a slight effect on the fluoride release ability of such CaF-doped GICs due to the very high insoubility of CaF_2_ [79]. Likewise, addition of TiO_2_ (3–5 wt. %) nano particles (nTiO_2_) to GIC powder has been shown to improve mechanical properties and anti-bacterial effects of the set material [69,70].

A few studies have attempted to evaluate the in vitro toxicity of modified GICs. nTiO_2_-containing GICs have been seen to stimulate the production of inflammatory factor prostaglandin E2 comparable to unmodified GICs [80]. However, more extensive studies are needed to ascertain the safety of nTiO_2_-containing GICs because free nTiO_2_ have been strongly suggested to be cytotoxic [81].

## 3. Nano-Filled Resin-Modified Glass Ionomer Cements

Unlike conventional GICs that consist of a glass powder and a polyacid solution, resin modified GICs also have a polymer resin component which usually sets by a self-activated (chemically cured) or light-activated polymerization reaction. These “hybrid” materials have been developed to combine the mechanical properties of a resin composite with the anti-carious potential of GICs [82]. Indeed, it has been observed that RMGICs not only release fluoride but they also have flexural strength superior to those of conventional GICs, as well as lower solubility [82]. Early RMGICs underwent slight 3.4% to 11.3% expansion due to water sorption after 24 h of placement [83]. However, newer formulations have overcome this problem [82]. When compared to resin composites, conventional RMGICs still possess reduced mechanical properties including brittleness and inferior strength along with aesthetics [2,3]. In addition, RMGICs have a decreased fluoride release and higher creep relative to conventional powder-based ionomers [3]. To overcome these drawbacks, there have been attempts to incorporate nano-sized fillers and bioceramics particles to RMGICs [84,85]. The different properties of these nano-filled RMGICs are discussed below.

### 3.1. Bonding of Nano-RMGIC with Tooth Structure

RMGICs micromechanically bond to dentine through infiltration of the collagen network previously exposed by using a 10% polyacrylic acid pre-treatment, in combination with chemical bonding obtained by ionic interaction of carboxyl groups from the acid with calcium ions of remaining HAP crystals within the partially demineralized dentin and enamel [3,86,87]. Nano-filled RMGICs exhibit a similar bonding mechanism but there is minimal infiltration of resin tags into dentin which is indicative of more ionic bonding with tooth rather than micromechanical retention, much akin to conventional GICs [84]. A commercially available nano-filled RMGIC (Ketac N100/Ketac Nano; 3M ESPE, St. Paul, MN, USA), contains nanoclusters of silica fillers and is supplied with a primer (Ketac Nano Primer). This is applied onto dental hard tissues before application of the cement [84]. However, recent studies have shown that in terms of micro-tensile bond strength (µTBS), there is no significant difference between nano-filled RMGIC and conventional RMGICs [84]. It is well-known that over-drying dentine following etching procedures can lead to the collapse of collagen fibers. The water-wet bonding technique allows the dentinal surface to remain wet with water that prevents the collapse of the demineralized dentinal matrix that occurs after extensive air drying. Unfortunately, it is impossible to infiltrate collapsed collagen with resin adhesives or RMGICs; hence, debonding and restoration failure are always common in such circumstances [4]. However, the use of specific dentin pre-treatments with functional primers may prevent the collapse of dentinal collagen fibers and improve the bonding performance of resin adhesive as well as RMGICs [88,89]. The so-called smear layer represents a further obstacle that can impede proper bonding of restorative materials to dental substrates. It is a kind of film of tooth debris left behind on the tooth surface following tooth preparation [90]. On the other hand, the pH of functional primers is low enough to remove most of the smear layer and improve the chemical bond to dentin and enamel [85,91]. Similarly, it has been demonstrated that acid etching (37% phosphoric acid) can improve the shear bond strength of nano-filled RMGIC by removing the smear layer and increasing the surface energy [85,92,93]. However, it is important to consider that GICs and RMGICs contain relatively high-molecular-weight polycarboxylic acid-based polymers (*M*w: 8000-to-15,000) that are excluded from infiltration of the phosphoric acid decalcified dentine [35,94]. Given this, in phosphoric acid-etched dentine, the collagen network can remain unprotected and be exposed to hydrolytic degradation. Such aggressive dentine pre-treatments should not be adopted when using GIC-based materials [32,34,77,95], because their polyalkeonic polymers are excluded from permeating into dentine collagen [96].

Therefore, it is believed that an additional bonding step (an acidic functional primer) or the use of a polyacrylic acid etching agent during nano-filled RMGIC restorations may increase the overall bonding performance of such materials. Laser-etching has also been studied as a potential conditioning method for nano-filled RMGIC. However, no significant improvement has been observed in bond strength when compared to conventional methods [97].

A randomized clinical trial investigating the performance of nano-RMGIC compared to conventional GIC and resin composites showed that there is no difference in one year survival of either type of restoration [98]. However, nano-RMGIC has been found to possess inferior marginal integrity compared to conventional RMGIC. This may be due to the inferior bonding performance of nano-RMGIC when applied onto enamel rather than dentine [99].

### 3.2. Mechanical and Physical Properties of Nano-RMGICs

Similar to all other composite restorative materials, modification of the size and shape of filler particles in a GIC can influence its mechanical properties [39,100]. Generally, a smaller particle size and higher packing density of fillers improves compression strength and hardness of GICs, whereas larger particles can lead to a higher wear resistance [39]. RMGICs have higher flexural strength, tensile strength, and resistance to solubility compared to conventional GICs which could be due to the chemical bonding between glass particles and the resin phase [39,101]. However, even if using smaller particles (>5 µm) has revealed to improve mechanical properties remarkably [102], the challenge that remains prevalent even in RMGICs is the poor surface properties compared to resin composite restorations [3,101].

Conventional RMGICs exhibited relatively better flexural strength and fatigue limit than commercially available nano-RMGICs [79,103]. In addition, nano-RMGICs have been shown to perform the worst when mechanically tested upon acid challenge [79]. Considering the abundance of acidogenic microbiota, oral pH may fall as low as 4 [104]. Acidic environment may jeopardize the long-term survival rate of nano-RMGICs owing to the susceptibility of these materials to acid erosion. Furthermore, addition of nHAp, nFAp and nanofluorohydroxyapatite to RMGICs can lead to an increased surface area, due to an increased filler-loading that may lead to improvements in the mechanical properties and bonding strength to the tooth [105]. On the other hand, setting time of nHAp modified RMGICs increases in excess of 800 s, which is remarkably higher than the ISO standard requirement (90–480 s) [106]. Such an increase in the setting time may be due to possible interference of nano-particles with the polymerization of the resins. However, the exact cause is not yet well understood.

### 3.3. Surface Mechanical Properties of RMGICs

The aesthetic properties of dental resin composite materials have been radically improved due to the incorporation of glass or ceramic nano-particles which make the surface of such materials easier to polish and provides with a greater reflectance index [106,107]. Similarly, GICs that have a smaller filler size distribution exhibited a smoother surface and are also easier to polish and finish [102]. Although purely mechanical degradation (abrasion) produced by tooth brushing simulates surface wear of nano-RMGCs to a significantly lesser degree compared to conventional RMGICs, in vitro studies suggest that there is no statistical difference between the surface roughness and hardness of nano-RMGICs and conventional RMGICs after bacterial and chemical degradation [108]. Nevertheless, to date, studies have established that no matter what size of filler is present in the RMGICs, their surface roughness and hardness remain significantly lower than those of resin composites following bacterial, mechanical, and chemical degradation due to lower wear resistance and higher solubility of the former [108,109,110]. Therefore, it can be concluded that commercially available nano-RMGICs do not possess any substantial advantage or disadvantage, in terms of surface mechanical properties, compared to conventional restorative materials.

### 3.4. Fluoride Release from Nano-Ionomers

It has been well established that fluoride is released from glass ionomers [8,111,112,113,114,115,116]. At high concentrations, fluoride has been thought to reduce the rate of demineralization, enhance the remineralization process, inhibit the growth, and attachment of bacteria on tooth surfaces and impede the formation of a complex bacteria biofilm [117]. Because the fluoride ions in the set ionomer structure do not take part in the setting reaction, they are released into the surrounding environment via an ion exchange process. Furthermore, the glass ionomers can also absorb salivary fluoride and act as fluoride reservoirs capable of releasing the ions which may have the potential to inhibit caries formation [118]. However, to date, it has not been established whether the amounts of fluoride released from glass ionomer cements is sufficient to impede dental caries [115,116]. Indeed, several studies have suggested that the cumulative fluoride release from nano-RMGICs and conventional RMGICs are comparable with each other but still significantly lesser than conventional GICs [79,119,120]. Nevertheless, the exact amount of fluoride released by nano-RMGICs compared to other RMGICs and GICs is still debatable. It has been observed that, although there is a slightly increased fluoride release from nano-RMGICs at a pH of 4, cumulative fluoride released after 84 days and per specimen surface in a day remains comparable to those of conventional RMGICs [79]. So far, no long-term clinical studies assessing the secondary caries in teeth restored by nano-ionomers cements are available in literature. This said, it has still not been ascertained whether or not the anti-carious activity of these cements is any better than conventional GICs in the clinical scenario. So far, with the exception of CaF_2_-modified ionomers, no studies have focused on investigating the effect of nano-modification of the powder component of conventional ionomers.

## 4. Conclusions

Nano-modification of conventional GICs and RMGICs can be achieved by the incorporation of nano-sized fillers to RMGICs, reducing the size of the glass particles and introducing nano-sized bioceramics to the glass powder. Commercially available nano-filled RMGIC (Ketac Nano) does not hold any significant advantage over micro-filled RMGICs in terms of flexural strength and tensile strength. Bonding properties of nano-filled RMGIC are still a matter of concern. Conversely, recent advancements, like the introduction of nano-sized apatites, have not only improved the mechanical properties of conventional GICs, but have also enhanced fluoride release in vitro. By increasing the crystallinity of the set matrix, apatite crystals can make the set cement more stable and improve the bond strength with tooth structure. An increased fluoride release can help in reducing secondary caries around restorations. However, a problematic issue is the possibility of the failure of the glass-bioceramic interface which may affect the mechanical properties of the set cement. Moreover, very few studies focusing on the nano-modification of GIC have concentrated on effects they might have on the pulpal cells. Hence, more mechanical, biological studies, and eventually, clinical trials are needed and essential to ascertain the status of nano-modified GICs in clinical practice. On top of that, there is more to be learned about the effect of nano-modification of the powder component in glass ionomers and on their fluoride release. Perhaps surprisingly, none of the studies have focused on investigating this aspect to date.

## Figures and Tables

**Table 1:** Some powder modifications of glass ionomer cements and their reported properties. HA, hydroxyapatite; FA, fluorohydroxyapatite; FAS, fluoroaluminosilicate; TiO_2_, titanium oxide; ZrO_2_, zirconium oxide.

GIC Formulation			Mechanical Properties			
Liquid	Powder	Nano Filler Percentage and Size	Compressive Strength (MPa)	Tensile Strength (MPa)	Flexural Strength (MPa)	Reference (s)
Polyacrylic acid copolymer	Unmodified FAS glass	No nano fillers, glass size: 3.34–9.6 µm	161	11.8	14.8	[45]
Polyacrylic acid copolymer	FAS Glass + HA	5 wt. %, 100–200nm	178	19	31	[44]
Polyacrylic acid copolymer	FAS Glass + FA	5 wt. %, 100–200	179	23	33	[44]
Polymer of AA, NVP, IA (8:1:1)	FAS Glass + HA	5 wt. %, 100–200 nm	183.8	23.5	36	[45]
Polyacrylic acid copolymer	FAS Glass + TiO_2_	3%, size variable	176.27	‒	23.17	[69,70]
Polyacrylic acid copolymer	FAS Glass + HA/ZrO_2_	4 vol. %, particle dimension: 20 × 200 nm	176.30	12.67	‒	[52]
